# Epiprofin Transcriptional Activation Promotes Ameloblast Induction From Mouse Induced Pluripotent Stem Cells *via* the BMP-Smad Signaling Axis

**DOI:** 10.3389/fbioe.2022.890882

**Published:** 2022-06-21

**Authors:** Xinchao Miao, Kunimichi Niibe, Yunyu Fu, Maolin Zhang, Praphawi Nattasit, Yumi Ohori-Morita, Takashi Nakamura, Xinquan Jiang, Hiroshi Egusa

**Affiliations:** ^1^ Division of Molecular and Regenerative Prosthodontics, Tohoku University Graduate School of Dentistry, Sendai, Japan; ^2^ Department of Prosthodontics, Affiliated Stomatology Hospital of Guangzhou Medical University, Guangdong Engineering Research Center of Oral Restoration and Reconstruction, Guangzhou Key Laboratory of Basic and Applied Research of Oral Regenerative Medicine, Guangzhou, China; ^3^ Department of Prosthodontics, Shanghai Ninth People’s Hospital, Shanghai Jiao Tong University School of Medicine; College of Stomatology, Shanghai Jiao Tong University; National Center for Stomatology; National Clinical Research Center for Oral Diseases; Shanghai Key Laboratory of Stomatology; Shanghai Engineering Research Center of Advanced Dental Technology and Materials, Shanghai, China; ^4^ Division of Molecular Pharmacology and Cell Biophysics, Tohoku University Graduate School of Dentistry, Sendai, Japan; ^5^ Center for Advanced Stem Cell and Regenerative Research, Tohoku University Graduate School of Dentistry, Sendai, Japan

**Keywords:** ameloblast, BMP-smad signaling pathway, cell differentiation, epiprofin, induced pluripotent stem cells, transcriptional activation

## Abstract

The transcriptional regulation of induced pluripotent stem cells (iPSCs) holds promise for their directed differentiation into ameloblasts, which are usually lost after tooth eruption. Ameloblast differentiation is regulated by multiple signaling molecules, including bone morphogenetic proteins (BMPs). Epiprofin (Epfn), a transcription factor, is expressed in the dental epithelium, and epithelial Epfn overexpression results in ectopic ameloblast differentiation and enamel formation in mouse incisor, a striking phenotype resembling that of mice with deletion of follistatin (a BMP inhibitor). However, it remains unknown whether and how Epfn transcriptional activation promotes ameloblast induction from mouse iPSCs. Here, we generated doxycycline-inducible *Epfn*-expressing mouse iPSCs (Epfn-iPSCs). Ameloblasts, which are characterized by positive staining for keratin 14 and amelogenin and alizarin red S staining, were successfully derived from Epfn-iPSCs based on a stage-specific induction protocol, which involved the induction of the surface ectoderm, dental epithelial cells, and ameloblasts at stages 1, 2, and 3, respectively. *Epfn* activation by doxycycline at stages 2 and/or 3 decreased cell proliferation and promoted ameloblast differentiation, along with the upregulation of p-Smad1/5/8, a key regulator of the BMP-Smad signaling pathway. Gene analysis of the BMP-Smad signaling pathway-associated molecules revealed that *Epfn* activation decreased follistatin expression at stage 2, but increased *BMP2/4/7* expression at stage 3. Perturbations in the ameloblast differentiation process were observed when the BMP-Smad signaling pathway was inhibited by a BMP receptor inhibitor (LDN-193189). Simultaneous LDN-193189 treatment and *Epfn* activation largely reversed the perturbations in ameloblast induction, with partial recovery of p-Smad1/5/8 expression, suggesting that *Epfn* activation promotes ameloblast induction from mouse iPSCs partially by upregulating BMP-Smad activity. These results reveal the potential regulatory networks between Epfn and the BMP-Smad pathway and suggest that Epfn is a promising target for inducing the differentiation of ameloblasts, which can be used in enamel and tooth regeneration.

## 1 Introduction

Tooth loss is common in clinics and usually results from periodontal and carious diseases, fractures, injuries, and genetic diseases (Egusa et al., 2012b; Suzuki et al., 2021). With the development of regenerative dentistry, stem cell-based tooth regeneration offers a promising approach for the treatment of missing teeth (Niibe et al., 2017). Tooth formation results from sequential and reciprocal interactions between dental epithelial cells (DECs) and dental mesenchymal cells (Balic and Thesleff, 2015). DECs produce ameloblasts for enamel formation, whereas dental mesenchymal cells form the dentine-pulp complex and periodontal tissues (Egusa et al., 2012a). While dental mesenchymal stem cells are maintained in adult tissues, including the dental pulp and the periodontal ligament, ameloblasts are eliminated soon after tooth eruption (Bluteau et al., 2008; Ahmed et al., 2020). Therefore, there is an urgent need to identify alternative sources of ameloblasts.

Ameloblast differentiation is regulated by multiple signaling molecules, including Wnt/β-catenin, Shh, and TGF-β/bone morphogenetic proteins (BMPs) (O'Connell et al., 2012; Balic and Thesleff, 2015). For instance, epithelial β-catenin overexpression results in supernumerary teeth with proper ameloblast differentiation and enamel formation (Järvinen et al., 2006). Several BMPs, such as BMP2, BMP4, and BMP7, are expressed during ameloblast development and could induce ameloblast differentiation *ex vivo* (Aberg et al., 1997; Wang et al., 2004). The continuously growing mouse incisors constitute a valuable model to study the regulation of ameloblast differentiation due to the presence of DECs and thus continuous ameloblast differentiation and enamel formation. Specially, enamel is present only on the labile side of the mouse incisor, while the lingual side is enamel-free due to the lingual expression of follistatin (Fst), a BMP inhibitor (Wang et al., 2004). It is reported that epithelial Fst overexpression induces a lack of enamel formation on both sides of mouse incisors, while epithelial Fst deletion results in bilateral enamel formation in mouse incisors, highlighting the importance of BMP signaling in ameloblast development (Wang et al., 2004).

The activity of signal molecules is mediated by transcription factors, which activate a battery of genes and eventually determine ameloblast differentiation (Balic and Thesleff, 2015; Yoshizaki et al., 2020). Epiprofin (Epfn), a zinc-finger transcription factor from the Sp/KLF family, is specifically expressed in the dental epithelial lineage (Nakamura et al., 2004). Epfn knockout mice do not exhibit ameloblast differentiation and enamel formation (Nakamura et al., 2008), whereas epithelial Epfn overexpression results in bilateral ameloblast differentiation and enamel formation in mouse incisor, a striking phenotype resembling that of mice with Fst deletion (Nakamura et al., 2017). These results suggest that Epfn is a key regulatory factor during ameloblast development. However, although there is a default signaling network *in vivo* to provide the factors required for Epfn to exert its effects, it remains unknown whether *Epfn* transcriptional activation alone can promote ameloblast induction *in vitro* from other cells used for tooth regeneration and whether Epfn acts by regulating BMP signaling.

Induced pluripotent stem cells (iPSCs) can be reprogrammed from somatic cells and can form any of the three germ layer cell types, thus representing a promising cell source for regenerative dentistry (Takahashi and Yamanaka, 2006; Zhang and Yelick, 2021). Recently, we successfully established a three-stage protocol for ameloblast induction from mouse iPSCs using specific signaling molecules, which provided a platform for evaluating the stage-specific role of target genes during ameloblast differentiation (Miao et al., 2021). It is known that transcriptional regulation in iPSCs also provides a strategy for ameloblast induction from iPSCs (Yoshizaki et al., 2020). The *piggyBac* transposon system, a DNA-based vector, has been widely used in genomic engineering of mammalian cells for preclinical research as well as clinical application due to its safety, efficiency, and stability (Woodard and Wilson, 2015; Sandoval-Villegas et al., 2021). In the present study, we generated doxycycline (Dox)-inducible *Epfn*-expressing mouse iPSCs (Epfn-iPSCs) using the *piggyBac* transposon system. We hypothesized that *Epfn* activation in iPSCs may promote ameloblast induction, possibly by regulating the BMP signaling pathway. The study aimed to evaluate the stage-specific effects of *Epfn* activation on ameloblast induction in mouse iPSCs and determine the potential involvement of BMP signaling in association with *Epfn* activation.

## 2 Materials and Methods

### 2.1 Generation of Dox-Inducible Epfn-Expressing Mouse iPSC Line (Epfn-iPSCs)

This study was approved by the Center and Committee of Gene Research, Tohoku University (approval nos. 2017DnLMO-011 and 2020DnLMO-007). A gateway entry vector containing the coding sequence of human Epfn (GenBank: AK127850.1; sequence: 279-1409) was purchased from the National Institute of Technology and Evaluation (NITE; Tokyo, Japan). The PB-TAC-ERN (KW111) vector (All-in-One *piggyBac* transposon destination vector) and pCAG-PBase expression vector (KW158) were generously gifted by Dr. Knut Woltjen (Kyoto University, Japan) (Kim et al., 2016). *Epfn* cDNA was transferred to the PB-TAC-ERN vector to generate the transposon PB-Epfn via the leukemoid reaction. Mouse iPSCs were cultured on inactivated SNLP76.7-4 feeder cells in ES medium containing DMEM (Nacalai Tesque, Kyoto, Japan) with 15% fetal bovine serum (Thermo Fisher Scientific, Waltham, MA, United States ), 2 mM L-glutamine (Wako, Osaka, Japan), 1 × 10^–4^ M nonessential amino acids (Thermo Fisher Scientific), 1 × 10^–4^ M 2-mercaptoethanol (Thermo Fisher Scientific), and 0.5% penicillin/streptomycin (Wako) (Egusa et al., 2010). Dox-inducible mouse Epfn-iPSCs were generated using the Neon transfection system (Thermo Fisher Scientific), as previously reported (Miao et al., 2021). Clones of mouse Epfn-iPSCs with high mCherry expression in the presence of 1 μg/ml Dox (Sigma-Aldrich, St Louis, MO, United States ) were selected for the subsequent experiments.

The Dox-inducible gene expression system was shown to be dose-dependent (Kim et al., 2016). To optimize the Dox concentration, Dox at different concentrations (0, 0.02, 0.2, 1, and 2 μg/ml) was added to the culture medium, and Epfn expression was evaluated using mCherry expression, real-time polymerase chain reaction (PCR), and Western blotting after 24 h. The pluripotency of mouse Epfn-iPSCs relative to naïve mouse iPSCs was investigated by alkaline phosphatase staining, immunofluorescence staining of Nanog and stage-specific embryonic antigen-1 (SSEA-1), and gene expression analysis of endogenous SRY-box 2 (*Sox2*), octamer-binding transcription factor 4 (*Oct4*), and Nanog using semi-quantitative reverse transcription PCR (RT-PCR).

### 2.2 Stepwise Ameloblast Induction From Mouse Epfn-iPSCs

Mouse Epfn-iPSCs were guided toward the ameloblast lineage using a previously established three-stage induction protocol (Miao et al., 2021). Briefly, mouse Epfn-iPSCs were dissociated into single cells using trypsin-EDTA, following which 3.0 × 10^5^ iPSCs were added to each well in low-attachment 6-well plates (Thermo Fisher Scientific) in ES medium under seesaw shaking at 30 rpm and an angle at 8 for 2 days to form embryoid bodies (days 0–2). The embryoid bodies were collected by centrifugation at 500 rpm for 3 min, seeded on gelatin-coated plates, and cultured in ES medium supplemented with 5 μM SB431542 (SB43; Sigma-Aldrich) for 3 days to induce surface ectoderm formation (stage 1; days 2–5). Then, the cells were incubated in DEC medium (composed of Dulbecco’s modified Eagle’s medium/F12 (Thermo Fisher Scientific), 20 ng/ml epidermal growth factor (Wako), 25 ng/ml basic fibroblast growth factor (Wako), 1 × B27 supplement (Thermo Fisher Scientific), and 1% penicillin/streptomycin) (Chavez et al., 2014) supplemented with 12.5 ng/ml BMP4 (Peprotech, Rocky Hill, NJ, United States ), 1 μM all-trans retinoic acid (Wako), and 20 mM lithium chloride (Wako) for 5 days to facilitate DEC differentiation (stage 2; days 5–10). Finally, the cells were guided toward the ameloblast lineage (stage 3; days 10–17) by culturing in SF2-differentiation medium containing 10 ng/ml epidermal growth factor, 3 ng/ml transforming growth factor β1 (Peprotech), and 15 mM LiCl for 7 days. The SF2-differentiation medium contained α-MEM (Nacalai Tesque) supplemented with 10% fetal bovine serum (Thermo Fisher Scientific), 20 mM β-glycerophosphate (Thermo Fisher Scientific), 50 μg/ml ascorbic acid (Thermo Fisher Scientific), 0.1 μM calcitriol (Wako), 2 mM calcium chloride (Wako), and 1% penicillin/streptomycin (Tadaki et al., 2016; Miao et al., 2021). Ameloblast differentiation was evaluated by immunocytochemical staining of keratin 14 (KRT14) and amelogenin (AMGN), Alizarin Red S (ARS) staining, and gene expression analysis using RT-PCR and real-time PCR.

### 2.3 Stage-specific Epfn Activation During Ameloblast Induction From Mouse Epfn-iPSCs

To evaluate the stage-specific role of *Epfn* activation in stepwise ameloblast induction from mouse Epfn-iPSCs, Dox was added to the induction medium at optimized concentrations at the indicated stage, as mentioned in the Result part. In addition, 500 nM LDN-193189 (BMP pathway inhibitor; Sigma-Aldrich) (Yu et al., 2008) was added during the indicated stage to determine whether Epfn acts in a BMP pathway-dependent manner. Cell morphology analysis, mCherry expression analysis, WST-1 test, ARS staining and quantification, RT-PCR, Western blotting, and real-time PCR were performed to assess the role of Epfn activation.

### 2.4 Experiment Protocols

#### 2.4.1 Semi-Quantitative and Real-Time RT-PCR

Total RNA was extracted using TRIzol (Thermo Fisher Scientific) and purified by treating with DNase I (Thermo Fisher Scientific). First-strand cDNA was synthesized using a reverse transcription system (Promega, Madison, WI, United States ). For semi-quantitative RT-PCR, the target genes were amplified using Taq DNA polymerase (Promega) according to the manufacturer’s instructions. PCR products were electrophoresed on 2% agarose gels with ethidium bromide and visualized under a UV transilluminator (Dolphin-View 2, WEALTEC, Sparks, NV, United States ). The results of RT-PCR were quantified using Image J software and normalized to *GAPDH* and the Dox-minus group. Real-time RT-PCR was performed using the Thunderbird SYBR qPCR Mix (Toyobo, Osaka, Japan) on a StepOnePlus real-time PCR system (Thermo Fisher Scientific). Gene expression data were analyzed quantitatively using the comparative cycle time (ΔΔCT) method (Miao et al., 2017). The primers used are listed in Supplementary Table S1.

#### 2.4.2 Western Blotting

Cells were lysed using the Blue Loading Buffer Pack (Cell Signaling, Danvers, MA, United States ), and the proteins were subjected to sodium dodecyl sulfate-polyacrylamide gel electrophoresis and transferred to a polyvinylidene difluoride membrane (Bio-Rad, Hercules, CA, United States ). After blocking with 5% non-fat milk for 60 min, the membranes were treated overnight with a primary antibody against Epfn (Atlas Antibodies, Stockholm, Sweden), KRT14 [MA5-11599 (LL002), Thermo Fisher Scientific], AMGN (Abcam, Cambridge, MA, United States ), ameloblastin [AMBN; sc-50534 (M-300), Santa Cruz Biotechnology, Santa Cruz, CA, United States ], kallikrein-related peptidase 4 (KLK4; Bioworld Technology Inc. Louis Park, MN, United States ), Fst [sc-365003 (C-8), Santa Cruz Biotechnology], phosphorylated Smad1/5/8 (p-Smad1/5/8, Cell Signaling), or β-actin (Cell Signaling) at 4°C. Next, the membranes were treated with the corresponding horseradish peroxidase-labeled secondary antibodies [anti-mouse (sc-516102) and anti-rabbit (sc-2357), Santa Cruz Biotechnology] for 60 min at room temperature. The immunoblots were detected using an Immunostar Zeta kit (Wako). The results of Western blotting were quantified using Image J software and normalized to β-actin and the Dox-minus group.

#### 2.4.3 Alkaline Phosphatase Staining

Cells were washed with phosphate buffer saline, fixed with 10% neutral buffered formalin, and stained for 30 min using 120 mM Tris buffer (Sigma-Aldrich) containing 1.8 mM fast red TR (Sigma-Aldrich) and 0.9 mM naphthol AS-MX phosphate (Sigma-Aldrich) at 37°C.

#### 2.4.4 Immunofluorescence and Immunocytochemistry Staining

Cells were fixed with 10% neutral-buffered formalin for 15 min and permeabilized in 0.2% Triton X-100 for 10 min. For immunofluorescence staining, the cells were blocked with 5% bovine serum albumin for 30 min and treated overnight at 4°C with primary antibodies against Nanog (Cell Signaling) or SSEA-1 (bs-1702R, Bioss, Woburn, MA, United States ). The samples were then treated with an Alexa Fluor 488-conjugated secondary antibody (ab150073, Abcam) for 60 min at room temperature. A fluorescence microscope (Zeiss AxioVert A1, Jena, Germany) was used to observe the staining.

For immunocytochemical staining, following fixation and permeabilization, as described above, the cells were treated with 0.3% H_2_O_2_ for 30 min and then blocked with 5% bovine serum albumin for another 30 min. The cells were then treated overnight with primary antibodies against KRT14 (MA5-11599, Thermo Fisher Scientific) or AMGN (ab153915, Abcam) at 4°C. This was followed by treatment with horseradish peroxidase-labeled specific secondary antibodies [anti-mouse (sc-5161029) and anti-rabbit (sc-2357), Santa Cruz Biotechnology] for 60 min at room temperature. The staining pattern was visualized using a diaminobenzidine kit (Roche Diagnostics, Mannheim, Germany).

#### 2.4.5 ARS Staining and Quantification

After washing with PBS and fixation with 10% neutral buffered formalin, the cells were incubated for 20 min with 40 mM ARS (Sigma-Aldrich) under gentle shaking. After washing four times with distilled water, the samples were observed under a microscope (Nikon, Tokyo, Japan) and scanned with the ApeosPort-VI C2271 (Fuji Xerox, Tokyo, Japan). For quantification, the ARS stain was dissolved in 10% acetic acid and neutralized with 10% ammonium hydroxide, as described previously (Wang et al., 2015). The extracted supernatant was analyzed by measuring absorbance at 405 nm using an iMark microplate reader (Bio-Rad).

#### 2.4.6 WST-1 Test

WST-1 solution (Roche, Germany) was diluted at a 1:10 ratio in an induction medium at the indicated stage. Absorption was measured at 450 nm using an iMark microplate reader (Bio-Rad) after 60 min of incubation with the cells.

### 2.5 Statistical Analysis

Quantitative results are expressed as mean ± standard deviation (*n* = 3). Unpaired *t*-test was used for comparison between two groups, with the Dox-minus group as a control. For three or more than three-group comparisons, one-way ANOVA (Tukey’s test) was performed to detect significant differences between each group and every other group. All statistical analyses were performed using the GraphPad Prism statistical software package (version 8.0), and differences were considered statistically significant at *p* < 0.05.

## 3 Results

### 3.1 Generation of Mouse Epfn-iPSCs

**FIGURE 1:**
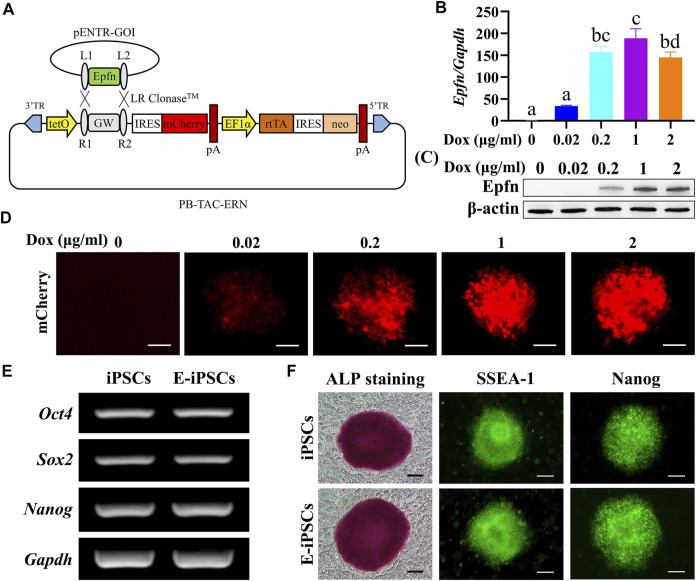
Establishment of mouse Epfn-iPSCs. **(A)** Generation of doxycycline-inducible Epfn-expressing *piggyBac* vector (PB-Epfn). **(B–D)** Inducible Epfn expression in Epfn-iPSCs after 24 h of culture with different doses of Dox (0–2 μg/ml) was examined by real-time PCR **(B)** and Western blot **(C)** along with mCherry expression **(D)**. **(E,F)** Epfn-iPSCs (E-iPSCs) showed comparable pluripotency to naive iPSCs according to the expression of pluripotent marker genes **(E)**, ALP staining, and immunofluorescence results for SSEA-1 and Nanog **(F)**. Scale bar: 100 μm.

The Dox-inducible *Epfn*-expressing *piggyBac* vector with a reporter gene (mCherry) is shown in Figure 1A. We observed that 1 μg/ml Dox induced the plateaued expression of Epfn and mCherry (Figures 1B–D) and was thus selected for *Epfn* activation in the subsequent experiments. In addition, Epfn-iPSCs expressed pluripotency markers at levels comparable with those of naïve iPSCs, as indicated by the expression of endogenous stem cell markers (*Oct4*, *Sox2*, and *Nanog*) (Figure 1E), alkaline phosphatase staining, and protein expression of SSEA-1 and Nanog (Figure 1F). These results suggest that we successfully generated a Dox-inducible *Epfn*-expressing mouse Epfn-iPSC line.

### 3.2 Ameloblast Differentiation From Epfn-iPSCs According to the Three-Stage Induction Protocol

**FIGURE 2:**
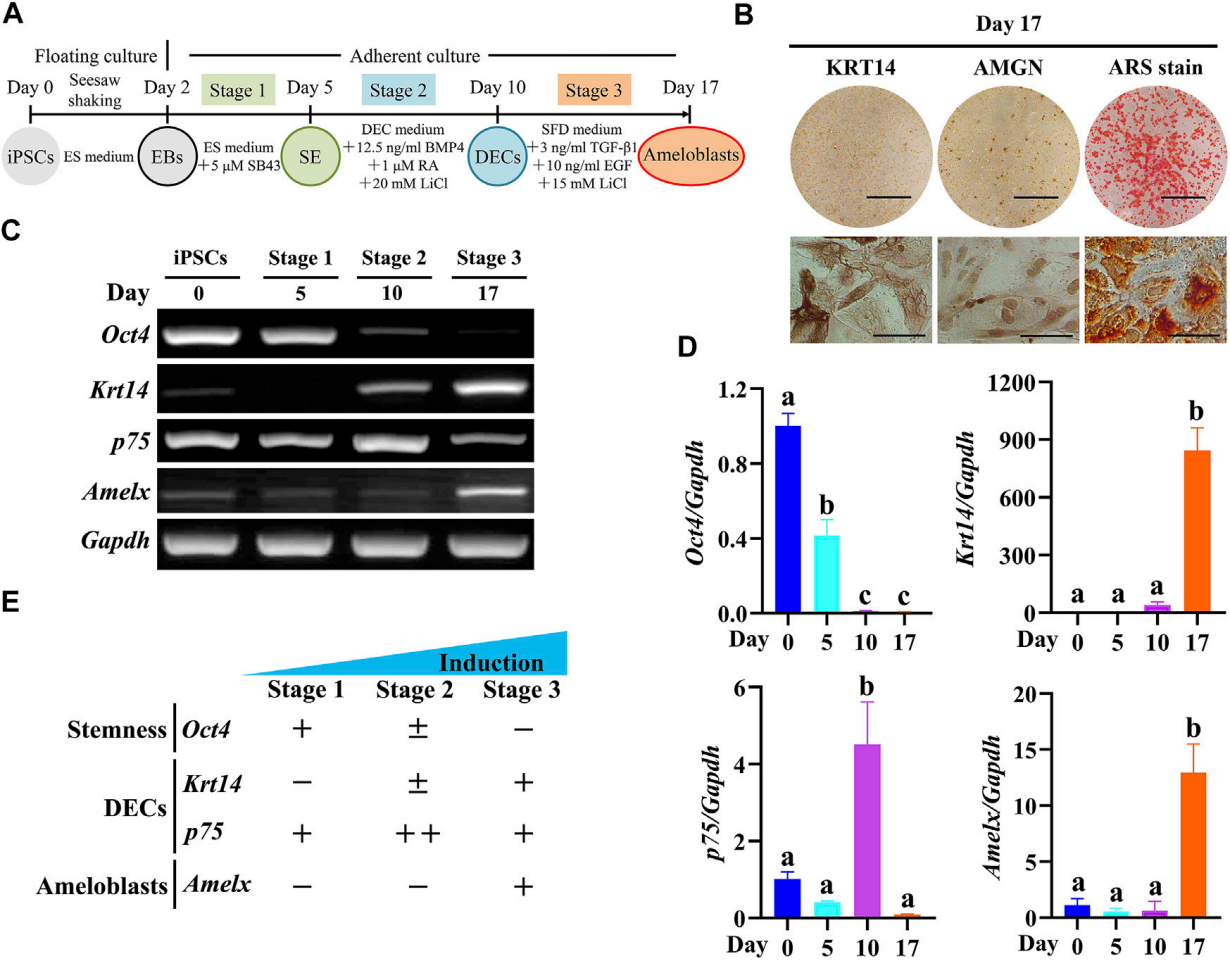
Induced ameloblast lineages from Epfn-iPSCs following a three-stage induction protocol. **(A)** Diagram of the three-stage ameloblast induction protocol. **(B)** Stain of keratin 14 (KRT14), amelogenin (AMGN), and Alizarin Red S (ARS) on day 17 after ameloblast induction. Scale bars: 1 cm and 100 μm for upper and lower panels, respectively. **(C,D)** Stage-specific marker gene expression during the stepwise ameloblast induction of Epfn-iPSCs by RT-PCR **(C)** and real-time PCR **(D)**. Different letters among groups (e.g., **A,B**) in Figure D indicate significant differences (*p* < 0.05; one-way ANOVA and Tukey’s test; *n* = 3). **(E)** Summary of gene expression in Figure C and D.

In a previous study, we established a three-stage stepwise ameloblast induction protocol using mouse Dox-inducible Amelx-iPSCs (Miao et al., 2021). Here, we confirmed whether Epfn-iPSCs could adopt an ameloblast fate after being treated according to the protocol (Figure 2A). After three-stage induction (day 17), the Epfn-iPSC-derived cells showed the expression of ameloblast markers (KRT14 and AMGN) and ARS staining, a phenotype of the ameloblast lineage (Figure 2B). We also evaluated gene expression patterns in each stage. The expression of *Oct4*, a stemness marker (Takahashi and Yamanaka, 2006), decreased markedly in the advanced stages (*p* < 0.05; Figures 2C,D). *Krt14*, a DEC marker (Kawano et al., 2004), was first expressed at stage 2 and was significantly upregulated at stage 3 (*p* < 0.05; Figures 2C,D). *p75*, a marker of inner enamel epithelial cells and pre-ameloblasts (Kawano et al., 2004), was transiently upregulated at stage 2 and downregulated at stage 3 when the cells were guided toward the ameloblast lineage (*p* < 0.05; Figures 2C,D). *Amelx*, an ameloblast marker (Gibson et al., 2001), only showed high expression at stage 3 (*p* < 0.05; Figures 2C,D). The gene expression pattern (Figure 2E) was in line with the *in vivo* expression during ameloblast development (Kawano et al., 2004), suggesting that Epfn-iPSCs were guided toward the ameloblast lineage when treated according to the established protocol.

### 3.3 Effect of Epfn Activation at Stage 1 on Surface Ectoderm Induction

*Epfn* is first expressed in the epithelium of developing teeth, hair follicles, and limb buds and in some ectodermal appendages (Nakamura et al., 2004). We determined whether *Epfn* activation could promote surface ectoderm differentiation at stage 1 (Supplementary Figure S1A). High *Epfn* expression was induced by Dox treatment, as indicated by the expression of the reporter gene mCherry (Supplementary Figure S1B) and *Epfn* transcripts (Supplementary Figure S1C); however, the cell morphology (Supplementary Figure S1B) and gene expression in the three germ layers (Supplementary Figure S1C) were highly comparable between the Dox-minus and Dox-plus groups, suggesting that *Epfn* activation might not affect cell differentiation at stage 1.

### 3.4 Effect of Epfn Activation at Stage 2 on DEC Induction

**FIGURE 3:**
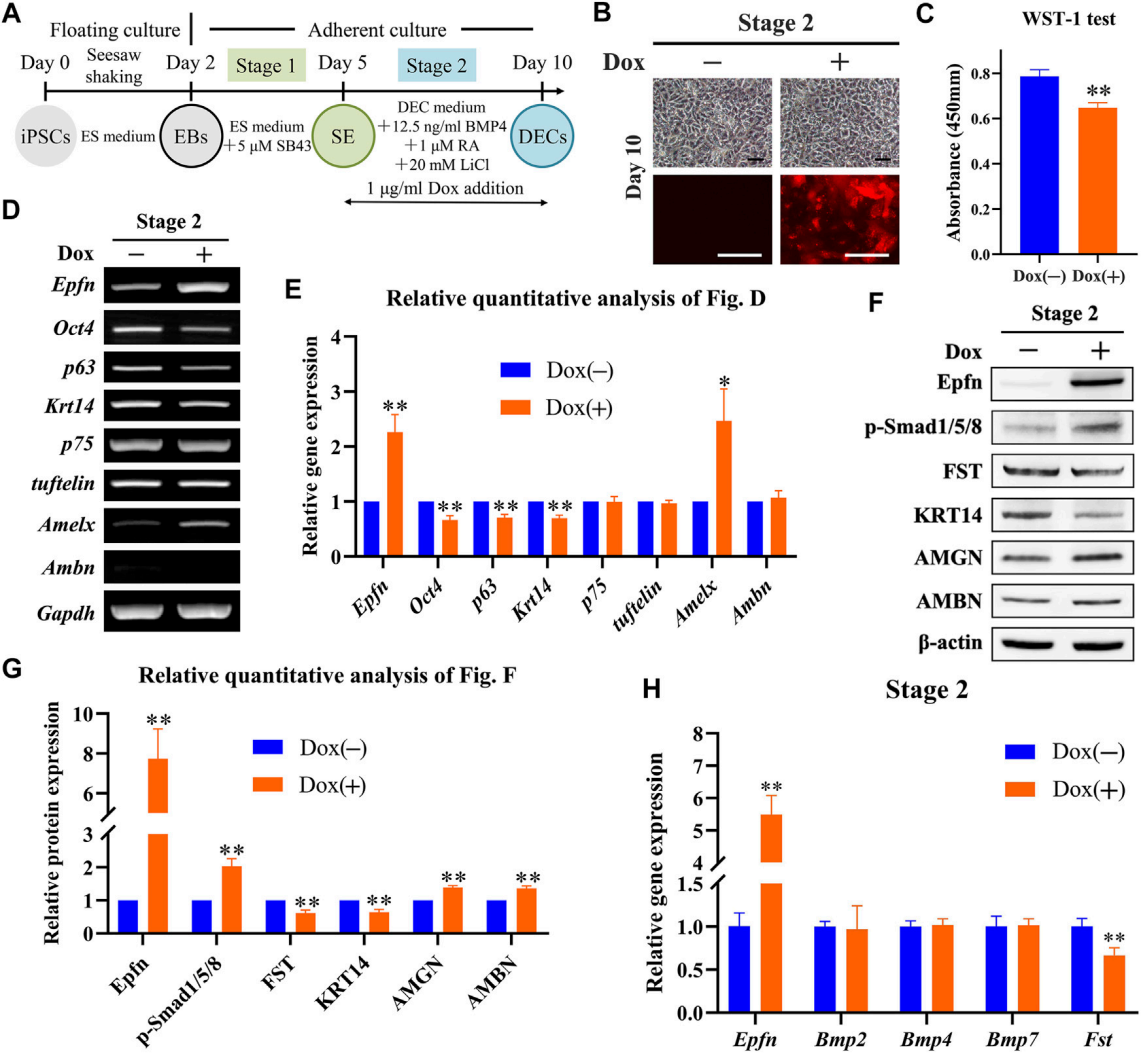
Role of Epfn activation at stage 2 in DEC induction. **(A)** Diagram of DEC induction from Epfn-iPSCs. Dox (1 μg/ml) was added at stage 2 to activate Epfn expression. **(B)** Cell morphology (upper) and mCherry expression (lower) on day 10. Note: the reporter gene mCherry was mainly located around the nucleus, with partial distribution in the cytoplasm. Scale bar: 100 μm. **(C)** WST-1 test on day 10. **, *p* < 0.01 (*t*-test; *n* = 3). **(D)** Gene expression according to RT-PCR analysis on day 10. Markers (stem cells: *Oct4*; proliferative epithelial: *p63*; dental epithelial: *Krt14*, *p75*, and *tuftelin*; and ameloblast: *Amelx* and *Ambn*). **(E)** Relative quantitative analysis of RT-PCR results. *, *p* < 0.05; **, *p* < 0.01 (*t*-test; *n* = 3) **(F)** Western blot on day 10. **(G)** Relative quantitative analysis of Western blot results. **, *p* < 0.01 (*t*-test; *n* = 3). **(H)** Real-time PCR of the BMP-Smad signaling pathway-associated molecules on day 10. **, *p* < 0.01 (*t*-test; *n* = 3).

We examined the effects of *Epfn* activation at stage 2 on DEC induction (Figure 3A). Dox treatment induced a high expression of the reporter gene mCherry, which was mainly located around the nucleus and partly in the cytoplasm; however, the cell morphology between the groups was similar (Figure 3B). *Epfn* overexpression has been reported to affect the proliferation and differentiation of DECs (Nakamura et al., 2011; Nakamura et al., 2017). Thus, we examined cell proliferation and differentiation after *Epfn* activation. The WST-1 test showed that *Epfn* activation significantly inhibited cell proliferation at stage 2 (*p* < 0.01; Figure 3C). The RT-PCR results showed that Dox-inducible *Epfn* activation upregulated *Amelx* transcripts but attenuated *Oct4*, *p63*, and *Krt14* expression (Figures 3D,E, *p* < 0.05). The groups showed similar levels of *p75*, *tuftelin*, and *Ambn* expression (Figures 3D,E). Western blotting revealed that Dox treatment led to high Epfn expression, enhanced AMBN and AMGN expression, but decreased KRT14 expression (Figures 3F,G, *p* < 0.01). Moreover, p-Smad1/5/8, a key regulator of the BMP-Smad pathway (Sartori et al., 2014), was upregulated, whereas FST, a BMP inhibitor (Wang et al., 2004), was downregulated (Figures 3F,G, *p* < 0.01). The BMP-Smad signaling pathway in DECs is regulated by BMP2/4/7 and FST (Wang et al., 2004). Here, we measured the gene expression of *Bmp2/4/7* and *Fst* to determine the mechanism underlying the upregulation of the BMP-Smad pathway after *Epfn* activation. Real-time PCR showed that Dox treatment significantly increased *Epfn* expression but decreased *Fst* expression (*p* < 0.01; Figure 3H), exerting barely any effect on *Bmp2/4/7* expression (Figure 3H). These results suggest that the upregulation of p-Smad1/5/8 might be caused by decreased FST expression. Taken together, the findings indicate that *Epfn* activation at stage 2 inhibited cell proliferation and promoted DEC differentiation into ameloblasts, besides upregulating the BMP-Smad signaling pathway.

### 3.5 Effect of Epfn Activation at Stage 3 on Ameloblast Induction

**FIGURE 4:**
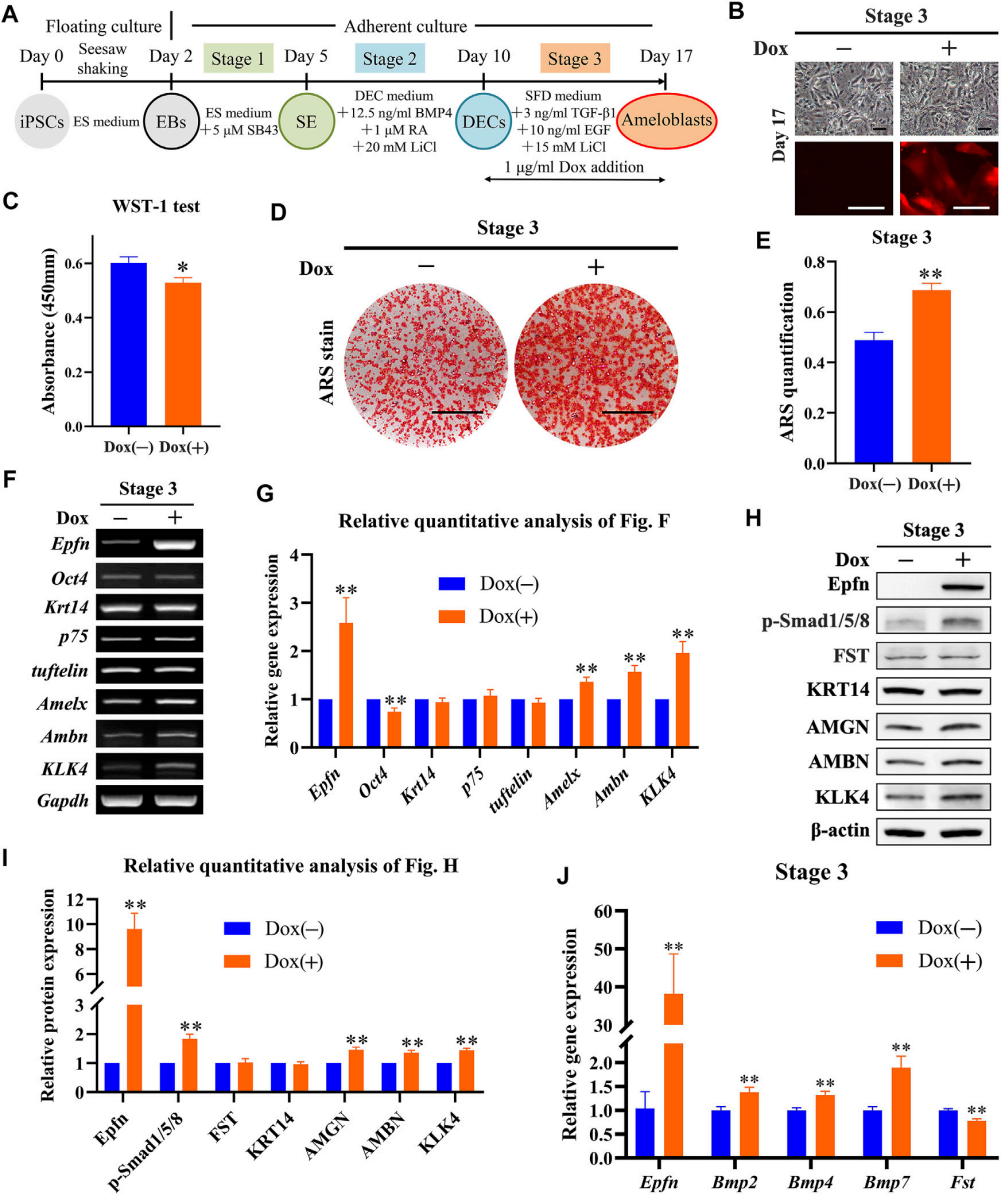
Role of Epfn activation at stage 3 in ameloblast induction. **(A)** Diagram of ameloblast induction from Epfn-iPSCs. Dox (1 μg/ml) was added at stage 3 to activate Epfn expression. **(B)** Cell morphology (upper) and mCherry expression (lower) on day 17. Scale bar: 100 μm. **(C)** WST-1 test on day 17. *, *p* < 0.05 (*t*-test; *n* = 3). **(D)** ARS stain on day 17. Scale bar: 1 cm. **(E)** Quantification of ARS staining on day 17. **, *p* < 0.01 (*t*-test; *n* = 3). **(F)** Gene expression according to RT-PCR analysis on day 17. Markers (stem cells: *Oct4*; dental epithelial: *Krt14*, *p75*, and *tuftelin*; and ameloblast: *Amelx*, *Ambn* and *KLK4*). **(G)** Relative quantitative analysis of RT-PCR results. **, *p* < 0.01 (*t*-test; *n* = 3). **(H)** Western blotting on day 17. **(I)** Relative quantitative analysis of Western blot results. **, *p* < 0.01 (*t*-test; *n* = 3). **(J)** Real-time PCR of the BMP-Smad signaling pathway-associated molecules on day 17. **, *p* < 0.01 (*t*-test; *n* = 3).

Next, we investigated the effects of *Epfn* activation at stage 3 on ameloblast induction (Figure 4A). The expression of the reporter gene mCherry was successfully induced after Dox treatment at stage 3, whereas a similar cell morphology was observed between the groups (Figure 4B). The WST-1 test showed that *Epfn* activation at stage 3 also significantly inhibited cell proliferation (*p* < 0.05; Figure 4C). ARS staining showed the presence of more positive areas in the Dox-plus group than in the Dox-minus group (Figure 4D), with a significant difference between the groups (*p* < 0.01; Figure 4E). RT-PCR showed that Dox treatment at stage 3 induced high levels of *Epfn* expression (*p* < 0.01), upregulated the ameloblast markers *Amelx*, *Ambn*, and *KLK4* (*p* < 0.01), and downregulated *Oct4* expression (*p* < 0.01), with comparable expression of *Krt14*, *p75*, and *tuftelin* (Figures 4F,G). Western blotting revealed that Dox treatment at stage 3 induced high levels of Epfn expression and enhanced AMBN, AMGN, and KLK4 expression (Figures 4H,I, *p* < 0.01). p-Smad1/5/8 expression was also found to be elevated after *Epfn* activation at stage 3 (*p* < 0.01), whereas FST expression was similar between the groups (Figures 4H,I). Real-time PCR showed that Dox treatment significantly upregulated *Epfn* transcripts, besides increasing *Bmp2/4/7* and decreasing *Fst* expression (*p* < 0.01; Figure 4J). Collectively, the enhancement of p-Smad1/5/8 expression at stage 3 may have resulted from the increased expression of BMPs. Taken together, *Epfn* activation at stage 3 inhibited cell proliferation and promoted ameloblast differentiation and mineralization, along with elevating p-Smad1/5/8 expression.

### 3.6 Effect of Epfn Activation at Stages 2 and 3 on Ameloblast Induction

**FIGURE 5:**
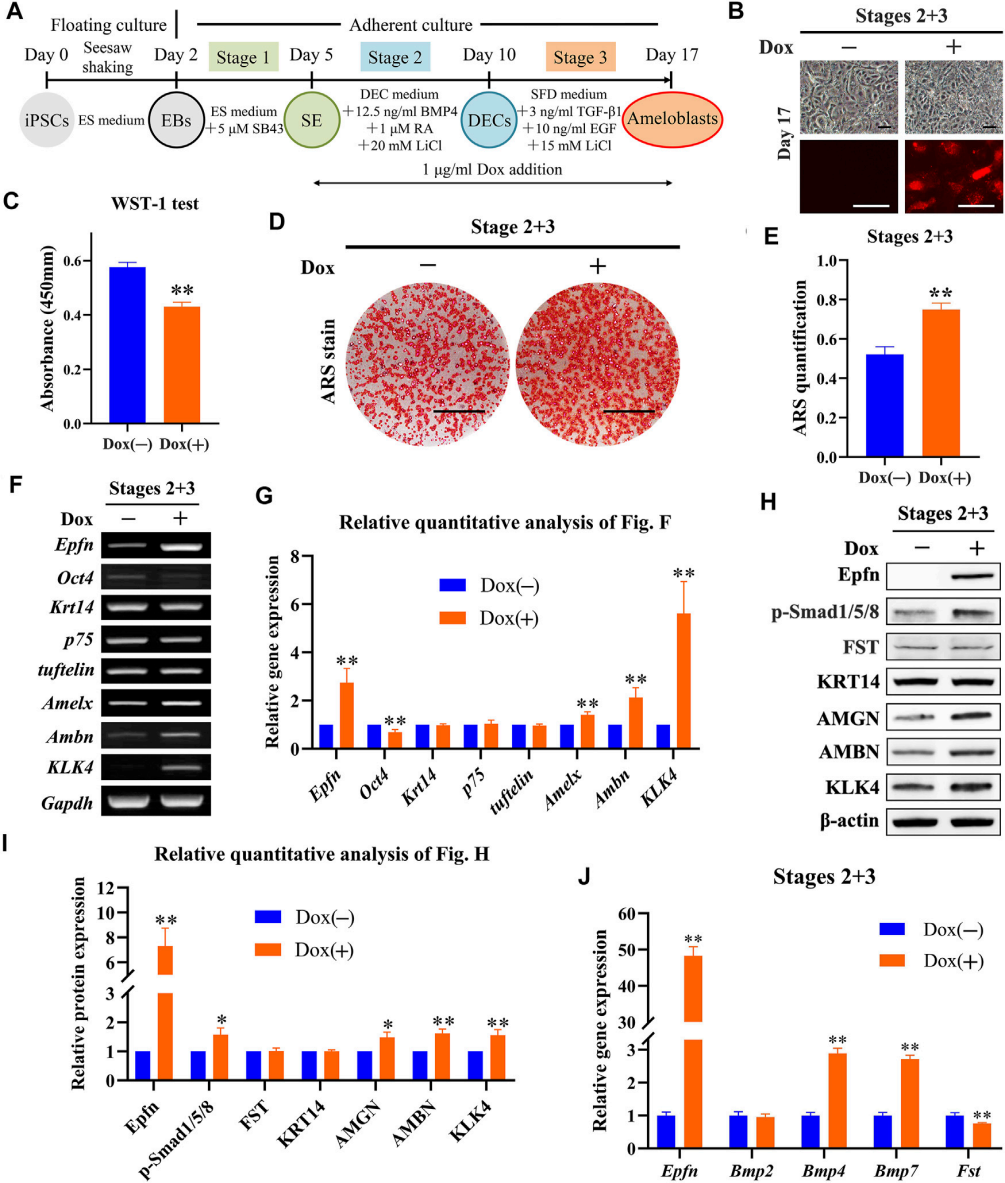
Role of Epfn activation at stages 2 and 3 in ameloblast induction. **(A)** Diagram of ameloblast induction from Epfn-iPSCs. Dox (1 μg/ml) was added at stages 2 and 3 to activate Epfn expression. **(B)** Cell morphology (upper) and mCherry expression (lower) on day 17. Scale bar: 100 μm. **(C)** WST-1 test on day 17. **, *p* < 0.01. **(D)** ARS stain on day 17. Scale bar: 1 cm. **(E)** Quantification of ARS staining on day 17. **, *p* < 0.01 (*t*-test; *n* = 3). **(F)** Gene expression according to RT-PCR analysis on day 17. Markers (stem cells: *Oct4*; dental epithelial: *Krt14*, *p75*, and *tuftelin*; and ameloblast: *Amelx*, *Ambn* and *KLK4*). **(G)** Relative quantitative analysis of RT-PCR results. **, *p* < 0.01 (*t*-test; *n* = 3). **(H)** Western blotting on day 17. **(I)** Relative quantitative analysis of Western blot results. *, *p* < 0.05; **, *p* < 0.01 (*t*-test; *n* = 3). **(J)** Real-time PCR of the BMP-Smad signaling pathway-associated molecules on day 17. **, *p* < 0.01 (*t*-test; *n* = 3).

Since Epfn is expressed from the dental placode stage to the ameloblast stage (Nakamura et al., 2004), which correspond to stages 2 and 3 in our study, respectively, we analyzed the effects of *Epfn* activation at stages 2 and 3 on ameloblast induction (Figure 5A). Although the reporter gene mCherry was induced after Dox treatment at stages 2 and 3, a similar cell morphology was observed between the groups (Figure 5B). *Epfn* activation during stages 2 and 3 significantly attenuated cell proliferation, as observed in the WST-1 test (*p* < 0.01; Figure 5C), but enhanced calcification in cells of the ameloblast lineage, as indicated by ARS staining and quantification (Figures 5D,E; *p* < 0.01). RT-PCR data revealed that *Epfn* overexpression at stages 2 and 3 was associated with the upregulation of *Amelx*, *Ambn*, and *KLK4* and downregulation of *Oct4* (*p* < 0.01), but induced no change in the expression of *Krt14*, *p75*, and *tuftelin* (Figures 5F,G). Western blotting revealed that Dox treatment at stages 2 and 3 promoted the expression of Epfn and ameloblast markers AMBN, AMGN, and KLK4 (*p* < 0.05), along with increasing the expression of p-Smad1/5/8 (*p* < 0.05) and slightly affecting the levels of expression of FST (Figures 5H,I). Real-time PCR revealed that Dox treatment significantly increased *Epfn* and *Bmp4/7* expression and decreased *Fst* expression (*p* < 0.01; Figure 5J). Interestingly, there was no significant difference in *Bmp2* expression between the groups after Epfn activation at stages 2 and 3 (Figure 5J). Taken together, *Epfn* activation at both stages 2 and 3 inhibited cell proliferation and promoted ameloblast differentiation and mineralization, and was also associated with elevated p-Smad1/5/8 expression, which was similar to the results observed with *Epfn* activation at stage 3.

### 3.7 Promotion of Ameloblast Differentiation by Epfn Partially via Upregulation of BMP-Smad Signaling

**FIGURE 6:**
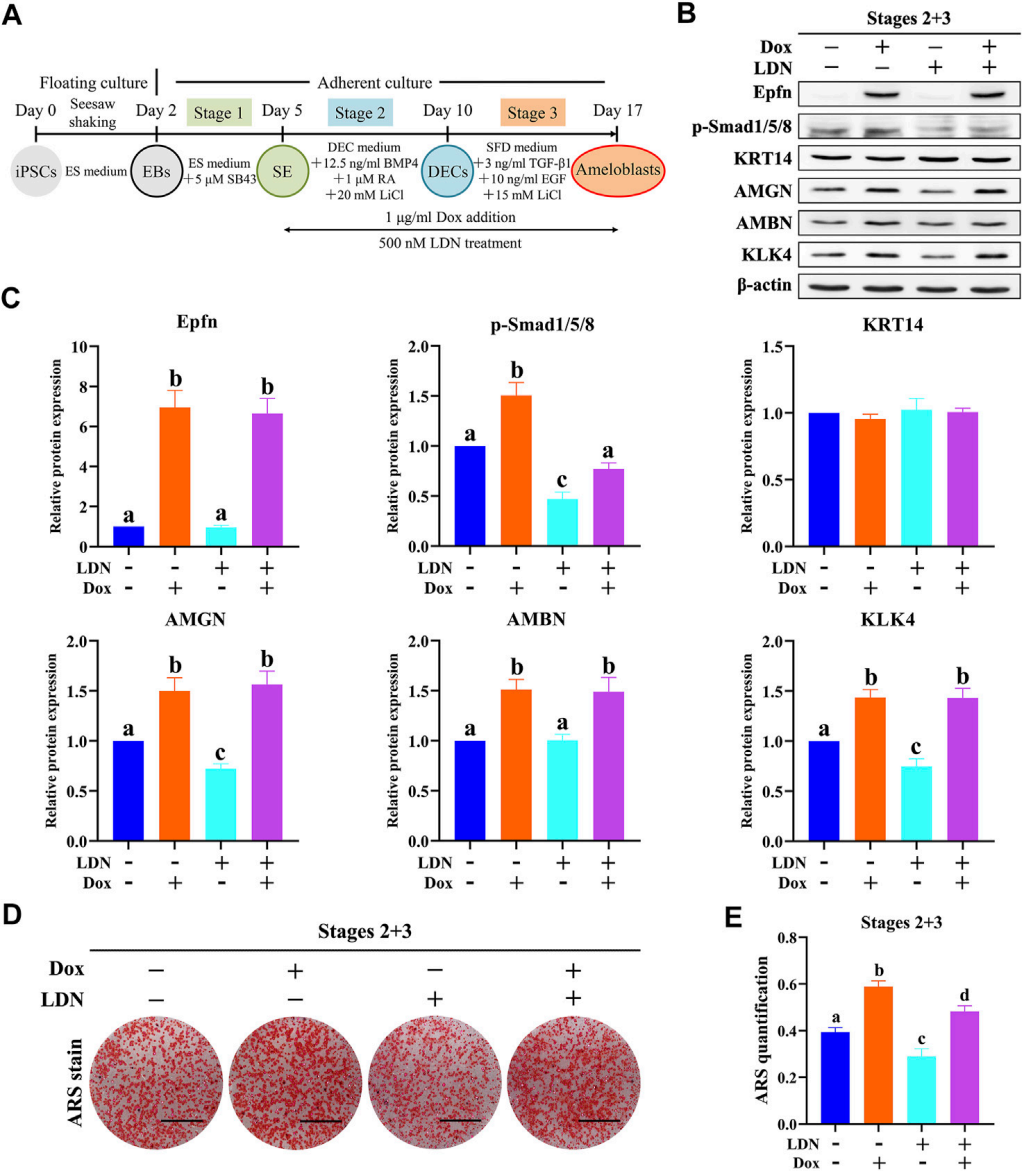
Epfn activation promotes ameloblast induction from mouse iPSCs partially by regulating the BMP-Smad pathway. **(A)** Diagram of ameloblast induction from Epfn-iPSCs. Dox (1 μg/ml) or 500 nM LDN-193189 (LDN, a BMP inhibitor) was added at stages 2 and 3 to activate Epfn expression or inhibit BMP-Smad signaling, respectively. **(B)** Western blotting on day 17. **(C)** Relative quantitative analysis of Western blot results. Different letters (e.g., **A,B**) indicate significant differences (*p* < 0.05; one-way ANOVA and Tukey’s test; *n* = 3). **(D)** ARS stain on day 17. Scale bar: 1 cm. **(E)** Quantification of ARS staining on day 17. Different letters (e.g., **A,B**) indicate significant differences (*p* < 0.05; one-way ANOVA and Tukey’s test; *n* = 3).

Exogenous BMP2/4/7 is known to induce ameloblast differentiation of enamel organs at the early bell stage, whereas a deletion of Fst (a BMP inhibitor) in epithelial cells is known to induce enamel formation in both the liable and lingual sides, suggesting that the BMP pathway acts as an inducer of ameloblast differentiation (Wang et al., 2004). *Similarly, epithelial Epfn overexpression in mice also caused bilateral enamel formation* (Nakamura et al., 2017). As our results showed that *Epfn* activation could concurrently promote ameloblast differentiation and BMP-Smad pathway upregulation, we investigated whether Epfn promotes ameloblast induction via upregulation of the BMP-Smad pathway. To test our hypothesis, we used LDN-193189, an inhibitor of the BMP receptor, to inhibit BMP-Smad signaling (Yu et al., 2008) (Figure 6A). LDN-193189 treatment caused a sharp decline in the expression of p-Smad1/5/8 and decreased the levels of the ameloblast markers AMGN and KLK4 (Figures 6B,C, *p* < 0.05). ARS staining and quantification showed significantly decreased calcification after LDN-193189 treatment (Figures 6D,E; *p* < 0.05). These results suggest that BMP-Smad signaling is required for proper ameloblast induction from mouse iPSCs. Interestingly, we found that simultaneous LDN-193189 treatment and *Epfn* activation via Dox treatment induced the expression of ameloblast markers (AMGN, AMBN, and KLK4) at levels similar to those observed in the *Epfn* activation group, and also partially restored the expression of p-Smad1/5/8 (Figures 6B,C). However, ARS staining and quantification showed that *Epfn* activation with simultaneous LDN-193189 treatment significantly promoted mineralization compared to LDN-193189 treatment alone, but the effect was still less than that of *Epfn* activation alone, indicating that the BMP-Smad pathway is required for proper ameloblast differentiation induced by Epfn activation (Figures 6D,E; *p* < 0.05). Taken together, these results suggest that the BMP-Smad pathway may partially account for the enhanced ameloblast differentiation induced in response to *Epfn* activation. In other words, *Epfn* promotes ameloblast differentiation from mouse iPSCs partially by upregulating the BMP-Smad pathway.

## 4 Discussion

Transcriptional regulation of pluripotent stem cells holds promise for their differentiation into cells of specific lineages, such as ameloblasts, which ceases to exist after tooth eruption (Zhang et al., 2016; Yoshizaki et al., 2020). Ameloblast differentiation is regulated by several conserved signaling molecules, including BMPs, and some transcription factors, such as Epfn (Balic and Thesleff, 2015). It is reported that Epfn knockout mice have no enamel (Nakamura et al., 2008), whereas epithelial Epfn overexpression results in bilateral ameloblast differentiation and enamel formation in mouse incisor, a striking phenotype resembling that of mice with deletion of Fst, a BMP inhibitor (Wang et al., 2004; Nakamura et al., 2017). However, it remains unknown whether and how *Epfn* activation promotes ameloblast induction from iPSCs. In the present study, we generated Dox-inducible *Epfn*-expressing iPSCs using the DNA-based *piggyBac* transposon system, which has been widely used in genomic engineering of mammalian cells for preclinical research and several clinical trials (Woodard and Wilson, 2015; Sandoval-Villegas et al., 2021). We found that *Epfn* activation in response to Dox treatment could promote ameloblast differentiation from mouse iPSCs, while decreasing cell proliferation and upregulating BMP-Smad activity, as indicated by enhanced p-Smad1/5/8 expression. Moreover, we found that BMP pathway inhibition by LDN-193189 treatment attenuated p-Smad1/5/8 and partially blocked ameloblast induction, whereas simultaneous LDN-193189 treatment and Epfn activation partially rescued the BMP-Smad pathway and largely promoted ameloblast differentiation. To the best of our knowledge, this is the first study to report the establishment of Dox-inducible Epfn-iPSCs and show that *Epfn* activation promotes ameloblast induction from mouse iPSCs, partially by upregulating BMP-Smad activity. Our results suggest that *Epfn* is a promising target gene for enhancing the induction of ameloblasts used in regenerative dentistry and provide insight into the potential regulatory networks between Epfn and the BMP-Smad pathway.

Epfn is expressed in epithelial cells during the early development of limbs, genitalia, and teeth during mouse embryo development (Nakamura et al., 2004). In developing teeth, Epfn is initially expressed in DECs at the placode and bud stages, following which its expression becomes restricted to IEEs and ameloblasts (Nakamura et al., 2004). Surprisingly, Epfn transgenic mice driven by the cytokeratin 5 promoter show ectopic ameloblast differentiation and enamel formation at both the labial and lingual sides of incisors, whereas enamel is normally absent from the lingual side of wild-type mouse incisor (Nakamura et al., 2017). These findings suggest that Epfn may direct the fate of DECs to ameloblasts and seems to be a promising target gene for promoting ameloblast induction from mouse iPSCs. We thus generated Dox-inducible mouse Epfn-iPSCs, which allowed for the stage-specific activation of *Epfn* under Dox treatment to mimic developmental expression. Recently, we established a three-stage ameloblast induction protocol from mouse iPSCs, which involved surface ectoderm induction (stage 1), DEC induction (stage 2), and ameloblast induction (stage 3) (Miao et al., 2021). Following the same protocol, we found that the Epfn-iPSC-derived cells stained positively for KRT14, AMGN, and ARS, and that the stage-specific gene expression pattern was consistent with the *in vivo* results; this indicated that our protocol is suitable for ameloblast induction from Epfn-iPSCs. We then evaluated the stage-specific role of *Epfn* activation in response to Dox treatment in ameloblast induction using the established protocol. *Epfn* activation promoted ameloblast induction at stages 2 and/or 3, as indicated by the upregulation of ameloblast makers (AMBN, AMGN, and KLK4) (Gibson et al., 2001; Fukumoto et al., 2004; Simmer et al., 2009) and ARS staining results. This is consistent with the findings of a previous study showing that Epfn overexpression in the rat DEC line SF2 promoted the expression of ameloblast markers, such as *Ambn* and *KLK4* (Nakamura et al., 2017). However, Epfn activation exerted a limited effect on surface ectoderm induction at stage 1, possibly owing to the fact that *Epfn* was not expressed in the surface ectoderm during development (Nakamura et al., 2004). Taken together, our results showed that *Epfn* activation alone could promote differentiation into ameloblasts from Epfn-iPSCs in a stage-specific manner.

BMP2/4/7 is known to induce ameloblast differentiation *ex vivo*, and epithelial overexpression of Fst, a BMP inhibitor, induces bilateral ameloblast differentiation and enamel formation in mouse incisor, whereas enamel is only present at the labial side of wild-type mouse incisor (Wang et al., 2004). Since epithelial Epfn overexpression also causes bilateral ameloblast differentiation and enamel formation in mouse incisor (Nakamura et al., 2017), we determined whether *Epfn* activation promoted ameloblast induction from iPSCs by regulating the BMP-Smad pathway. Interestingly, we found that BMP-Smad activity, reflected by p-Smad1/5/8 expression (Sartori et al., 2014; Omi et al., 2020), was upregulated after Epfn activation at stages 2 and 3. However, the mechanisms underlying the enhanced BMP-Smad pathway appeared to differ between stages 2 and 3. *Epfn* activation at stage 2 downregulated FST expression but did not alter *Bmp* expression, whereas *Epfn* activation at stage 3 promoted *Bmp2/4/7* expression, with limited changes in FST expression. These results suggest that increased BMP-Smad activity was primarily attributed to decreased FST expression at stage 2 and increased *BMP* expression at stage 3. The different mechanisms underlying enhanced BMP activity following *Epfn* activation suggest that *Epfn* may cooperate with other molecules to regulate BMP activity at different developmental stages. We further attempted to determine whether the upregulation of BMP-Smad activity induced by Epfn overexpression promoted ameloblast induction after Epfn overexpression. LDN-193189, a small molecule, was demonstrated to be a specific BMP inhibitor targeting BMP type I receptors, thus preventing the signaling transduction from extracellular BMP ligands, including BMP2/4/7, that were upregulated by Epfn activation during ameloblast induction at stage 3 (Horbelt et al., 2015). We observed that the BMP-Smad pathway was partially blocked in response to LDN-193189 treatment, resulting in disrupted ameloblast induction as indicated by AMGN and KLK4 downregulation and decreased ARS staining, indicating that the BMP-Smad pathway is required for proper ameloblast induction from mouse iPSCs. Interestingly, simultaneous Epfn activation and LDN-193189 treatment induced the expression of the ameloblast markers AMGN, AMBN, and KLK4 at almost the same levels as those in the group subjected to only Epfn activation, along with the partial rescue of the BMP-Smad pathway. However, the staining intensity of ARS in the group with simultaneous *Epfn* activation and LDN-193189 treatment was weaker than that in the group with *Epfn* activation alone. Taken together, these results suggest that the BMP-Smad pathway partially accounts for the enhanced ameloblast induction caused by Epfn activation. In other words, our results showed that *Epfn* activation promoted ameloblast induction when BMP signaling was partially blocked, which may explain the phenotype of enamel formation on the lingual side of incisors in Epfn-overexpressing mice, in which Epfn overexpression abolished the inhibitory effect of Fst exerted at the lingual side on the BMP signaling pathway. It is reported that non-Smad pathways, including p38, ERK1/2, and Akt, could also be inhibited by LDN-193189 (Boergermann et al., 2010; Calpe et al., 2016), and whether these pathways are also responsible for Epfn-induced ameloblast differentiation requires further research.

We also found that *Epfn* activation at stages 2 and 3 decreased cell proliferation to a moderate degree. Reportedly, Epfn exerts distinct effects on DEC proliferation: transient Epfn expression stimulates cell proliferation, whereas stable Epfn expression inhibits cell proliferation (Nakamura et al., 2011). In our cell model, we used the *piggyBac* transposon system, which allows the stable expression of Epfn. Our results are consistent with those of a previous report (Nakamura et al., 2011). It is well known that tooth morphogenesis is regulated by a balance between cell proliferation and differentiation (Nakamura et al., 2017). The slightly decreased proliferation induced by Epfn overexpression could not only ensure a sufficient number of cells required for the bioengineered teeth (Ikeda et al., 2009), but also allowed fine-tuning of cell proliferation to obtain an optimized shape of the tooth crown, which is indispensable for clinical application. It should be noted that the mechanism underlying the process by which stable *Epfn* activation inhibits DEC proliferation remains unknown.

The effect of Epfn overexpression in promoting ameloblast induction from mouse iPSCs was significant but moderate. It is reported that Wnt/β-catenin signaling lies upstream of the signaling network during odontogenesis and could promote the expression of Epfn, Shh, FGF, and BMP4 (Aurrekoetxea et al., 2016). During DEC induction at stage 2 and ameloblast induction at stage 3, LiCl, a Wnt/β-catenin activator, was used and shown to promote cell differentiation in a concentration-dependent manner (Miao et al., 2021). Since LiCl could strongly promote ameloblast induction, the continuous presence of LiCl may overshadow the effectiveness of Epfn overexpression on cell differentiation to a certain degree. Another report found that enamel matrix genes, including Amelx, Ambn, and KLK4, contained Epfn target sequences (Rhodes et al., 2021). Our results demonstrated that Epfn activation could promote the expression of these ameloblast markers to a certain extent. As it is known, Epfn cooperates with other factors to initiate the expression of target genes. However, in our system, only Epfn was overexpressed, which may limit the effects of Epfn overexpression on ameloblast induction due to the relative lack of other factors. The slight promotion of ameloblast differentiation from iPSCs by Dox-inducible stage-specific Epfn activation, together with the moderate inhibition of cell proliferation by Epfn, suggests that Epfn seems to be a promising target for fine-tuning the regulation of morphogenesis during enamel formation. The *in vivo* behavior of Dox-inducible Epfn overexpression in optimizing the size of the tooth crown needs further investigation, which is conducive to regenerative dentistry regarding enamel and tooth regeneration.

In conclusion, this is the first study to establish a Dox-inducible Epfn-iPSC model for the stage-specific evaluation of *Epfn* activation during ameloblast induction. Following the induction protocol, we found that *Epfn* activation promoted ameloblast differentiation and decreased cell proliferation to a moderate extent, which suggests that Epfn may be a novel target allowing for fine-tuning of morphogenesis during enamel formation. Moreover, we elucidated, for the first time, that Epfn enhanced ameloblast differentiation partially via upregulation of the BMP-Smad pathway. These results suggest that Epfn is a promising target for ameloblast induction, which is used in regenerative dentistry. However, further research is needed on the mechanism underlying the process by which Epfn expression enhances ameloblast differentiation and decreases cell proliferation.

## Data Availability

The raw data supporting the conclusions of this article will be made available by the authors, without undue reservation.

## Abbreviations

iPSCs, induced pluripotent stem cells; BMP, bone morphogenetic protein; Epfn, epiprofin; Epfn-iPSCs, *Epfn*-expressing mouse induced pluripotent stem cells; DEC, dental epithelial cell; Fst, follistatin; Dox, doxycycline; PCR, polymerase chain reaction; RT-PCR, reverse transcription polymerase chain reaction; KRT14, keratin 14; AMGN, amelogenin; ARS, Alizarin Red S; AMBN, ameloblastin; KLK4, kallikrein-related peptidase 4; p-Smad1/5/8, phosphorylated Smad1/5/8.
