# Supplementary material for: Epiprofin Transcriptional Activation Promotes Ameloblast Induction From Mouse Induced Pluripotent Stem Cells via the BMP-Smad Signaling Axis
Source: Front Bioeng Biotechnol. 2022 Jun 21;10:890882. doi: 10.3389/fbioe.2022.890882 (PMC9253510; doi:10.3389/fbioe.2022.890882)
Supplement: Supplementary file 1 [file DataSheet1.docx]

Supplementary Material

**Supplementary Figure and Table**


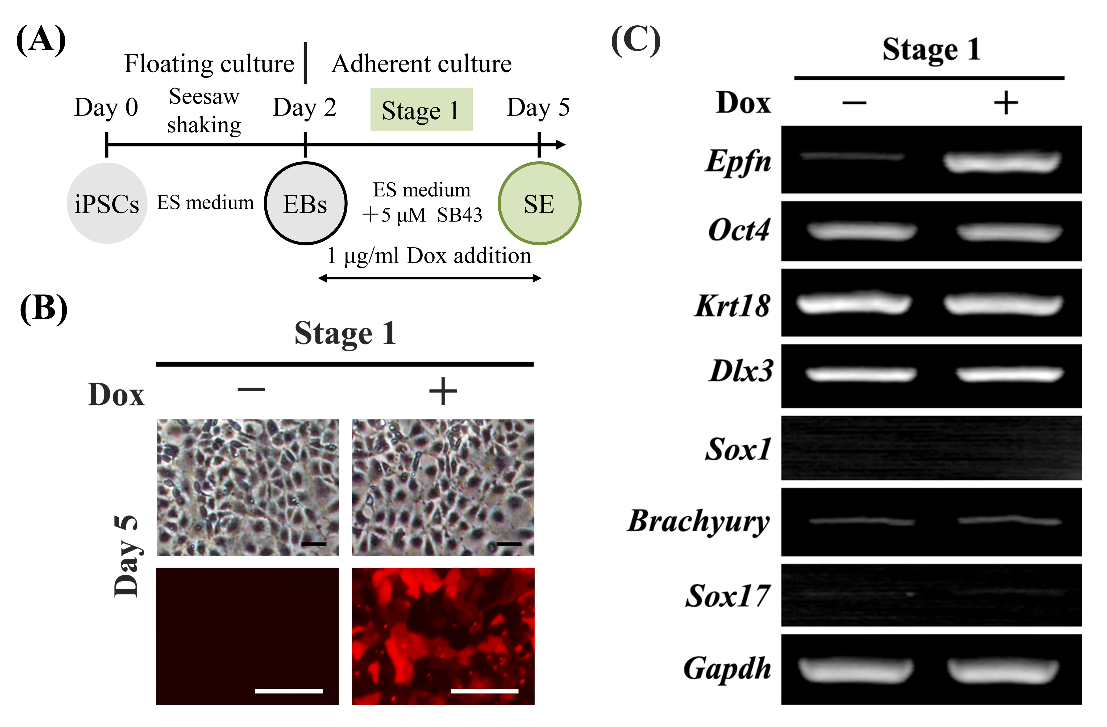


**Supplementary Figure 1. Role of Epfn activation at stage 1 in SE induction.**

(A) Diagram of SE induction from Epfn-iPSCs. 1 μg/mL Dox was added at stage 1 to activate Epfn expression. (B) Cell morphology (upper) and mCherry expression (lower) on day 5. Scale bar: 100 μm. (C) Evaluation of three germ layer markers by RT-PCR on day 5. Markers (stem cell: Oct4, SE: Krt18, non-neural ectoderm: Dlx3, neural ectoderm: Sox1, mesoderm: Brachyury, endoderm: Sox17.)

**Supplementary Table 1. Primers used for reverse transcription-polymerase chain reaction (RT-PCR) and real-time RT-PCR**.

| Gene | Forward primer | Reverse primer | Size (bp) |
| --- | --- | --- | --- |
| 1. mouse primers for RT-PCR | | | |
| *Oct4* | TCTTTCCACCAGGCCCCCGGCTC | TGCGGGCGGACATGGGGAGATCC | 224 |
| *Sox2* | TAGAGCTAGACTCCGGGCGATGA | TTGCCTTAAACAAGACCACGAAA | 297 |
| *Nanog* | AGGGTCTGCTACTGAGATGCTCTG | CAACCACTGGTTTTTCTGCCACCG | 364 |
| *Krt18* | AGATCGACAATGCCCGCCTT | TGCAGAAGGACCCCATTGAGC | 574 |
| *Dlx3* | TCTGGTTCCAGAACCGCCGCT | TCAGTACACAGCCCCAGGGTTA | 341 |
| *Sox1* | CGGATCTCTGGTCAAGTCGG | GGGACCTCGGTACAAAGTCG | 340 |
| *Brachyury* | CCAGCTCTAAGGAACCACCG | TGTCCACGAGGCTATGAGGA | 450 |
| *Sox17* | TCTGCACAACGCAGAGCTAA | GCATAGTCCGAGACTGGAGC | 504 |
| *p63* | GGAAAACAATGCCCAGACTC | GTGGAATACGTCCAGGTGGC | 294 |
| *Krt14* | ACCAAAGGCCGTTACTGCAT | GAGGAGAATTGAGAGGATGAGGA | 233 |
| *p75* | ATGGAAGGGGACCGAGATGA | CCGGTGGGGTCCTTCTACTA | 571 |
| *tuftelin* | TCAGCCGTTATCAGCGAGAAG | AGTCAGCGTTCTTGATCCGAA | 198 |
| *Ambn* | TCACCCCTGAATTAGCAGAAGT | GCTCTTGGAAACGCCATGC | 174 |
| *Amelx* | TGACTCCAACCCAACACCAT | GCTTGGTCTTGTCTGTCGCT | 246 |
| *KLK4* | GCTGCTGTATGACCCTGTGT | GTGGGCCTTGTAGTCAGTCC | 253 |
| *GAPDH* | CACCATGGAGAAGGCCGGGG | GACGGACACATTGGGGGTAG | 418 |
| 2. mouse primers for real-time RT-PCR | | | |
| *Oct4* | *AGAGGATCACCTTGGGGTACA* | *CGAAGCGACAGATGGTGGTC* | *96* |
| *Krt14* | *ACCAAAGGCCGTTACTGCAT* | *GAGGAGAATTGAGAGGATGAGGA* | *233* |
| *p75* | *CTAGGGGTGTCCTTTGGAGGT* | *CAGGGTTCACACACGGTCT* | *140* |
| *Amelx* | CCCCAGTCACCTCTGCATC | GCTGCATGGAGAACAGTGG | 73 |
| *Bmp2* | GGGACCCGCTGTCTTCTAGT | TCAACTCAAATTCGCTGAGGAC | 154 |
| *Bmp4* | TTCCTGGTAACCGAATGCTGA | CCTGAATCTCGGCGACTTTTT | 114 |
| *Bmp7* | ACGGACAGGGCTTCTCCTAC | ATGGTGGTATCGAGGGTGGAA | 164 |
| *Fst* | CTCCTCAAGGCCAGATGCAA | TGGAGCTGCCTGGACAAAAA | 100 |
| *GAPDH* | TGCACCACCAACTGCTTAG | GGATGCAGGGATGATGTTC | 177 |
| 3. human primers for RT-PCR | | | |
| *Epfn* | CTCGTGGTGGGACCTTCATC | AATGCTTCTTCTTGCCCCCA | 404 |
| 4. human primers for real-time RT-PCR | | | |
| *Epfn* | GGACTTCTCGCAGGGCTATG | GCTGCAGGAGCTTGGAAAAG | 110 |
